# Effect of oromotor stimulation on early sucking behavior and later development in preterm newborns

**DOI:** 10.6026/973206300213749

**Published:** 2025-10-31

**Authors:** Ankit Jain, Sardar Vikram Singh Bais, Akash yadav, Kamini Goyal

**Affiliations:** 1 Department of Pediatrics, Shyam Shah Medical College and Associated Gandhi Memorial hospital, Chakaraghat, Sagar, Madhya Pradesh, India; 2 Department of Pediatrics Birsa Munda Govermemt Medical College, Shahdol, Madhya Pradesh, India; 3 Department Pediatrics, Shrimant Rajmata Vijayaraje Scindhiya Medical College, Shivpuri, Madhya Pradesh, India; 4 Department of Paediatrics, NSCB Medical College and Hospital, Jabalpur, Madhya Pradesh, India

**Keywords:** Oromotor stimulation, preterm infants, sucking behavior, nutrition, development, oral feeding

## Abstract

Preterm infants (<34 weeks) often face feeding difficulties due to immature oromotor coordination. This randomized controlled trial of 150 preterm infants compared structured OMS, unstructured stimulation and standard care. Standard care led to earlier full oral feeding (8.84 ± 2.37 days), greater weight gain (40.04 ± 6.92%) and shorter hospital stay (24.16 ± 4.52 days) (p < 0.001). Developmental scores at 6 months were significantly higher with standard care than with OMS interventions. Standard care proved more effective than OMS in enhancing feeding, growth and neurodevelopmental outcomes in preterm infants.

## Background:

Preterm birth, defined as birth before 37 completed weeks of gestation, remains a significant global health challenge. Worldwide, approximately 10.6% of all live births are preterm, with India reporting a higher prevalence of around 13.61% [1]. Advances in neonatal care have improved survival rates of preterm infants, particularly those born at earlier gestational ages. However, these infants often face significant challenges in achieving oral feeding competence due to immature oromotor coordination [2]. Feeding difficulties in preterm infants stem from the incomplete development of sucking, swallowing and breathing coordination, which typically matures around 34 weeks of gestation [3]. The earliest literature on neonatology by Dr. Pierre Budin in 1890 identified feeding problems as a primary concern for preterm babies [4]. Successful oral feeding relies on the proper maturation of these functions, with jaw movements becoming visible as early as the 11th week of intrauterine life [5]. However, the coordination of sucking, swallowing and breathing is not fully developed until approximately 34 weeks of gestation, making infants born before this period particularly vulnerable to feeding difficulties [6]. Preterm infants often require prolonged tube feeding, which can negatively impact their ability to transition to full oral feeding and may lead to feeding disorders and oral hypersensitivity [7, 8]. Achieving full oral feeding is essential for hospital discharge, making the development of effective interventions to facilitate this transition a priority in neonatal care [9, 10]. Various strategies have been employed to enhance oral feeding performance in preterm infants, including non-nutritive sucking, modified positioning, pacing and oral motor stimulation [11]. Oromotor stimulation (OMS) refers to structured manipulative actions targeting the lips, jaw, tongue and soft palate before feeding, aimed at enhancing the maturation of sucking skills [12]. The use of a pacifier in preterm infants has been shown to significantly shorten the time required to achieve full oral feeding, establish exclusive breastfeeding, and attain hospital discharge, demonstrating its beneficial role in neonatal feeding development [13]. Similarly, father-infant skin-to-skin contact (SSC) in the neonatal intensive care unit has proven feasible and is being explored for its potential to enhance neurodevelopmental, physiological, and paternal psychological outcomes in moderately preterm infants [14]. In addition, the application of Premature Infant Oral Motor Intervention (PIOMI) has been shown to improve feeding progression and strengthen oral motor skills in preterm neonates, thereby supporting more effective feeding transitions [15]. Consistent with these findings, the regular use of a pacifier has been further associated with improved sucking ability and a shorter duration to reach full breastfeeding and hospital discharge, emphasizing the importance of early oral stimulation techniques in preterm infant care [16, 17]. Despite these promising findings, there remains a gap in understanding the comparative effectiveness of different OMS protocols versus standard care. Most studies have focused on comparing OMS interventions to standard care without differentiating between structured and unstructured approaches [18]. Furthermore, the optimal protocol for OMS, including timing, duration and frequency of intervention, requires further investigation [19]. Therefore, it is of interest to evaluate and compare the effectiveness of structured and unstructured oromotor stimulation with standard care in improving feeding efficiency, nutritional outcomes and developmental progress among preterm infants.

## Materials and Methods:

## Study design and setting:

A prospective, randomized controlled trial was conducted in the Neonatal Intensive Care Unit (NICU) of a tertiary care teaching hospital in central India between January 2021 and December 2023. The study was approved by the Institutional Ethics Committee and written informed consent was obtained from the parents or legal guardians of all participants before enrollment.

## Participants:

Preterm infants with gestational age <34 weeks admitted to the NICU were eligible for inclusion. Infants with major congenital anomalies, severe intraventricular hemorrhage (Grade III-IV), periventricular leukomalacia, necrotizing enterocolitis, hemodynamic instability, or those requiring mechanical ventilation at the time of enrollment were excluded from the study.

## Sample size:

The study included 150 preterm infants, with 50 infants randomly assigned to each of three intervention groups. This sample size was determined based on previous studies and was adequate to detect clinically significant differences in primary outcomes with 80% power and 5% significance level.

## Randomization and blinding:

Eligible infants were randomly assigned to one of three groups using computer-generated block randomization: Group A (structured OMS), Group B (unstructured oral stimulation), or Group C (standard care). The randomization sequence was concealed in sealed opaque envelopes. Due to the nature of the intervention, the therapists administering the stimulation could not be blinded. However, the outcome assessors and data analysts were blinded to the group allocation.

## Intervention protocol:

Group A: Structured Oromotor Stimulation

Infants in this group received structured OMS based on the Premature Infant Oral Motor Intervention (PIOMI) protocol.

The intervention consisted of eight steps performed in sequence:

[1] Cheek and lip stroking (1 minute)

[2] Jaw stabilization (1 minute)

[3] Tongue stimulation (1 minute)

[4] Palatal stimulation (1 minute)

[5] Gum massage (1 minute)

[6] Cheek and chin support (1 minute)

[7] Non-nutritive sucking with pacifier (2 minutes)

[8] Sucking-swallowing-breathing coordination exercises (2 minutes)

The intervention was administered for 10 minutes, twice daily (morning and evening), starting at 32 weeks postmenstrual age (PMA) until full oral feeding was achieved. All interventions were performed by trained neonatal therapists who had completed a standardized training program on the PIOMI protocol.

## Group B: Unstructured oral stimulation:

Infants in this group received unstructured oral stimulation for the same duration (10 minutes, twice daily) as Group A. The intervention consisted of gentle stroking around the oral area without following a specific protocol or sequence. The stimulation was performed by the same therapists who administered the structured OMS to ensure consistency in contact time.

## Group C: Standard care:

Infants in this group received standard care as per the NICU protocol, which included routine positioning, non-nutritive sucking with pacifier during gavage feeding and gradual transition to oral feeding based on clinical assessment. No additional oral stimulation was provided.

## Outcome measures:

## Primary outcomes:

[1] Time to achieve full oral feeding: Defined as the number of days from the initiation of oral feeding attempts until the infant could sustain full oral feeding (120 mL/kg/day) without the need for gavage supplementation.

[2] Weight gain percentage: Calculated as the percentage increase in weight from birth to discharge.

[3] Length of hospital stay: Defined as the number of days from birth until discharge.

## Secondary outcomes:

[1] Nutritional outcomes at 6 months corrected age: Assessed using anthropometric measurements including weight, length, head circumference and mid-upper arm circumference (MUAC).

[2] Neurodevelopmental outcomes at 6 months corrected age: Assessed using the Developmental Assessment Scale for Indian Infants (DASII), which provides a Developmental Motor Quotient (DMoQ) and Developmental Mental Quotient (DMeQ), expressed as percentages.

## Data collection:

Baseline data including gestational age, birth weight, sex, mode of delivery, Apgar scores and maternal characteristics were collected at enrollment. Daily feeding records, including type of feeding (gavage, oral, or mixed), volume of intake and feeding tolerance, were maintained. Weight was measured daily using an electronic infant scale with a precision of 5 grams. Length and head circumference were measured weekly using standardized techniques. Follow-up assessments were conducted at 6 months corrected age in the developmental follow-up clinic. Anthropometric measurements were performed by trained nurses and neurodevelopmental assessments were conducted by a developmental pediatrician blinded to the group allocation.

## Statistical analysis:

Data were analyzed using SPSS version 25.0 (IBM Corp., Armonk, NY, USA). Normality of data distribution was assessed using the Shapiro-Wilk test. Continuous variables were expressed as mean ± standard deviation (SD). Categorical variables were expressed as frequencies and percentages. For intergroup comparisons, independent samples t-test was used for continuous variables. Chi-square test or Fisher's exact test was used for categorical variables as appropriate. A p-value <0.05 was considered statistically significant. Intention-to-treat analysis was performed, including all randomized participants in the groups to which they were originally assigned.

## Results:

A total of 150 preterm infants were included in the study, with 50 infants randomly assigned to each group. All participants completed the intervention period and 130 (86.7%) completed the 6-month follow-up (44 in Group A, 44 in Group B and 42 in Group C). Twenty participants (13.3%) were lost to follow-up due to relocation or inability to contact. The baseline characteristics of the participants are presented in Table 1 (see PDF). There were no significant differences among the three groups in terms of gender, locality, maternal education, parity, history of abortion, number of siblings, type of delivery, resuscitation, malformation, or feeding method (p>0.05 for all comparisons). However, some differences were noted in neonatal variables between groups B and C, including age at admission (p=0.0493), age of starting intervention (p=0.0009) and age of starting feed (p=0.0010). Infants in Group C achieved full oral feeding significantly earlier (mean ± SD: 8.84 ± 2.37 days) compared to Group A (23.20 ± 16.80 days) and Group B (18.75 ± 12.76 days) (p<0.001). The difference between Group A and Group B was not statistically significant (p=0.2118) (Table 2 - see PDF). Weight gain percentage was significantly greater in Group C (40.04 ± 6.92%) compared to Group A (28.77 ± 9.71%) and Group B (34.14 ± 10.93%) (p<0.001). Group B also showed significantly greater weight gain than Group A (p=0.0170) (Table 2 - see PDF). Hospital stay was significantly shorter in Group C (24.16 ± 4.52 days) than in Group A (26.92 ± 5.17 days) (p=0.0055). The difference between Group B and Group C was not statistically significant (p=0.0714), nor was the difference between Group A and Group B (p=0.2765) (Table 2 - see PDF). At 6 months corrected age, infants in Group C had significantly higher weight (6.42 ± 0.21 kg) compared to Group A (5.65 ± 0.43 kg) and Group B (5.97 ± 0.37 kg) (p<0.001). Similarly, length was significantly greater in Group C (62.81 ± 2.09 cm) than in Group A (58.09 ± 2.62 cm) and Group B (60.73 ± 2.27 cm) (p<0.001). Head circumference was also significantly larger in Group C (41.95 ± 1.31 cm) compared to Group A (39.73 ± 1.56 cm) and Group B (40.64 ± 1.42 cm) (p<0.001). MUAC was significantly greater in Group C (12.12 ± 0.27 cm) than in Group A (11.74 ± 0.34 cm) and Group B (11.95 ± 0.28 cm) (p<0.001) (Table 3 - see PDF). At 6 months corrected age, Group C showed significantly higher developmental scores (DMoQ: 78.14 ± 5.59%; DMeQ: 72.81 ± 5.12%) compared to Group A (DMoQ: 64.50 ± 8.92%; DMeQ: 63.50 ± 9.15%) and Group B (DMoQ: 68.95 ± 8.89%; DMeQ: 65.32 ± 7.70%) (p<0.001 for both DMoQ and DMeQ). Group B also showed significantly higher DMoQ scores than Group A (p=0.0212), but the difference in DMeQ scores between Group A and Group B was not statistically significant (p=0.3162) (Table 3 - see PDF). Discharge rates were 88% in Group A, 92% in Group B and 96% in Group C, with the remaining participants leaving against medical advice (LAMA). The differences in discharge rates among the groups were not statistically significant (p=0.3145) (Table 4 - see PDF).

## Discussion:

The present study evaluated the effect of structured oromotor stimulation (OMS), unstructured oral stimulation and standard care on early sucking behavior, nutrition and development in preterm infants. Contrary to our hypothesis, the results demonstrated that standard care (Group C) was significantly more effective than both structured OMS (Group A) and unstructured stimulation (Group B) in improving feeding outcomes, weight gain and neurodevelopmental outcomes at 6 months corrected age. The primary outcome of time to achieve full oral feeding was significantly shorter in the standard care group compared to both intervention groups. Infants receiving standard care achieved full oral feeding 14.36 days earlier than those receiving structured OMS and 9.91 days earlier than those receiving unstructured stimulation. The discrepancy may be attributed to differences in the standard care protocols between studies or variations in the implementation of the OMS interventions. Weight gain percentage was significantly higher in the standard care group compared to both intervention groups. The superior weight gain in the standard care group may be related to the earlier achievement of full oral feeding, allowing for more efficient nutritional intake and reduced energy expenditure during feeding [20, 21-22]. The reduction in hospital stay observed in the standard care group has important clinical and economic implications. Infants receiving standard care were discharged 2.76 days earlier than those receiving structured OMS. The longer hospital stay in the intervention groups may be related to feeding difficulties or other complications associated with the OMS interventions [23, 24- 25]. The nutritional benefits observed at 6 months corrected age were particularly noteworthy. Infants in the standard care group had significantly higher weight, length, head circumference and MUAC compared to the intervention groups. These findings suggest that the benefits of standard care extend beyond the neonatal period and may have lasting effects on growth and nutrition. The improved nutritional outcomes in the standard care group may be attributed to the establishment of better feeding patterns during the critical neonatal period [26]. The neurodevelopmental outcomes at 6 months corrected age revealed significant benefits of standard care. Infants in the standard care group had higher developmental motor and mental quotients compared to the intervention groups. The mechanisms underlying the association between standard care and improved neurodevelopment may include reduced stress associated with feeding difficulties and improved nutritional status supporting brain development. Several factors may explain the superior outcomes in the standard care group. First, the standard care protocol in this study included non-nutritive sucking with pacifier during gavage feeding and gradual transition to oral feeding based on clinical assessment, which may have been more effective than the structured or unstructured OMS interventions. Second, the OMS interventions may have caused stress or discomfort to the infants, leading to poorer feeding outcomes. Third, the timing, duration, or frequency of the OMS interventions may not have been optimal for this population [27, 28]. The findings of our study have several important clinical implications. First, standard care protocols that include non-nutritive sucking and gradual transition to oral feeding may be more effective than structured or unstructured OMS for preterm infants. Second, the implementation of OMS interventions in neonatal care units should be carefully evaluated, as they may not provide additional benefits beyond standard care. Third, further research is needed to identify the optimal approach to facilitating oral feeding in preterm infants. Despite the strengths of our study, including its randomized controlled design, comprehensive assessment of outcomes and adequate sample size and several limitations should be acknowledged. First, the study was conducted at a single center, which may limit the generalizability of the findings. Second, the therapists administering the intervention could not be blinded to the group allocation, which may have introduced bias. Third, the follow-up period was limited to 6 months corrected age and longer follow-up would be needed to assess the persistence of the benefits. Fourth, the study did not evaluate the potential mechanisms underlying the differences between the groups. Future research should focus on identifying the components of standard care that are most effective for facilitating oral feeding in preterm infants. Additionally, studies investigating the optimal timing, duration and frequency of OMS interventions would be valuable. Finally, multicenter trials with larger sample sizes and longer follow-up periods are needed to confirm and extend our findings.

## Conclusion:

Standard care was significantly more effective than structured or unstructured oromotor stimulation in improving early sucking behavior, facilitating earlier transition to oral feeding, enhancing weight gain, reducing hospital stay and improving neurodevelopmental outcomes at 6 months in preterm infants. Thus, we show that the current standard care protocol may be superior to the tested OMS interventions for preterm infants and highlight the importance of carefully evaluating feeding interventions before implementation in clinical practice.

## References

[1] Li Y (2024). Trials..

[2] Li L (2021). Trials..

[3] Pickler RH (2015). Trials..

[4] Lessen BS (2011). Adv Neonatal Care..

[5] Ostadi M (2021). Int J Pediatr Otorhinolaryngol..

[6] Sun H (2020). Trials..

[7] Cresi F (2019). Trials..

[8] Shaki F (2022). BMC Pediatr..

[9] Muelbert M (2019). Cochrane Database Syst Rev..

[10] Yin LP (2015). Trials..

[11] Foster JP (2016). Cochrane Database Syst Rev..

[12] Aagaard H (2015). JBI Database Syst Rev Implement Rep..

[13] Say B (2018). Breastfeed Med..

[14] Deng Q (2018). Trials..

[15] Ghomi H (2019). Int J Pediatr Otorhinolaryngol..

[16] Kaya V (2017). J Clin Nurs..

[17] McFadden A (2021). Health Technol Assess..

[18] Li L (2022). J Pediatr (Rio J)..

[19] Yang C (2019). Zhonghua Wei Zhong Bing Ji Jiu Yi Xue..

[20] Celen R (2021). JPEN J Parenter Enteral Nutr..

[21] Dorling J (2020). Health Technol Assess..

[22] Huo J (2021). Trials..

[23] da Rosa Pereira K (2020). PLoS One..

[24] Beker F (2023). Trials..

[25] El-Kassas O (2024). BMC Pediatr..

[26] Alidad A (2021). J Pediatr Rehabil Med..

[27] Guler S (2022). Adv Neonatal Care..

[28] Neel ML (2019). BMC Pediatr..

